# Brainstem Infarction and Panuveitis due to Sarcoidosis Successfully Treated with Steroid Pulse Therapy

**DOI:** 10.1155/2012/356743

**Published:** 2012-02-08

**Authors:** Natsuyo Yoshida-Hata, Shigeko Yashiro, Noritoshi Arai, Sousuke Takeuchi

**Affiliations:** ^1^Department of Ophthalmology, National Center for Global Health and Medicine, Tokyo 162-8655, Japan; ^2^Department of Neurology, National Center for Global Health and Medicine, Tokyo 162-8655, Japan

## Abstract

A 36-year-old man visited our hospital because of blurred vision and redness of the conjunctiva. Slit-lamp examination showed panuveitis. Two days later, he suddenly experienced dizziness, speech disturbance, paralysis of his right extremities, and gait disturbances. Neurological examinations suggested that his symptoms were caused by a left lateral medullary lesion. He also had erythema mainly on his trunk. Magnetic resonance imaging (MRI) of his brain demonstrated a small infarct on the left side of the medulla oblongata. Clinical presentation and MRI findings were consistent with the diagnosis of a Wallenberg's syndrome. He also had bilateral hilar lymphadenopathy. A skin biopsy showed granulomatous nodular dermatitis compatible with sarcoidosis. He was treated with steroid pulse therapy and his neurological and ocular symptoms immediately improved. Only seven similar cases of intracranical sarcoidosis have been reported, but none had been treated with steroid pulse therapy. We recommend that steroid pulse therapy be considered to treat patients with sarcoidosis with signs of lesions in the central nervous system.

## 1. Introduction

Sarcoidosis is characterized by noncaseating granulomas, and in 5% of the patients, the lesions are located in the central nervous system [1]. However, strokes are uncommon in patients with sarcoidosis in spite of small vessel inflammation, and sarcoidosis in the medulla oblongata is rare [2–4]. Sarcoidosis is a systemic disease associated with uveitis and accounts for 10% of the uveitis cases in Japan [5, 6]. Ocular sarcoidosis is managed well by systemic corticosteroid therapy and is generally easier to manage than eyes with Bechet disease [7].

Chronic posterior ocular sarcoidosis is more difficult to manage, and it can lead to sight-threatening complications, for example, cataracts, glaucoma, and cystoid macular oedema. Eyes with posterior uveitis with chronic sarcoidosis have a poor visual prognosis despite immunosuppressive therapy [8].

Corticosteroids, usually combined with immnosuppresive agents, remain the mainstay therapy for ocular sarcoidosis although refractory cases do occur [9]. The refractory cases generally have a poor visual prognosis.

We described a patient with ocular sarcoidosis and a cerebral infarction, who had an excellent response to steroid-pulse therapy.

## 2. Case Report

**Figure 1:**
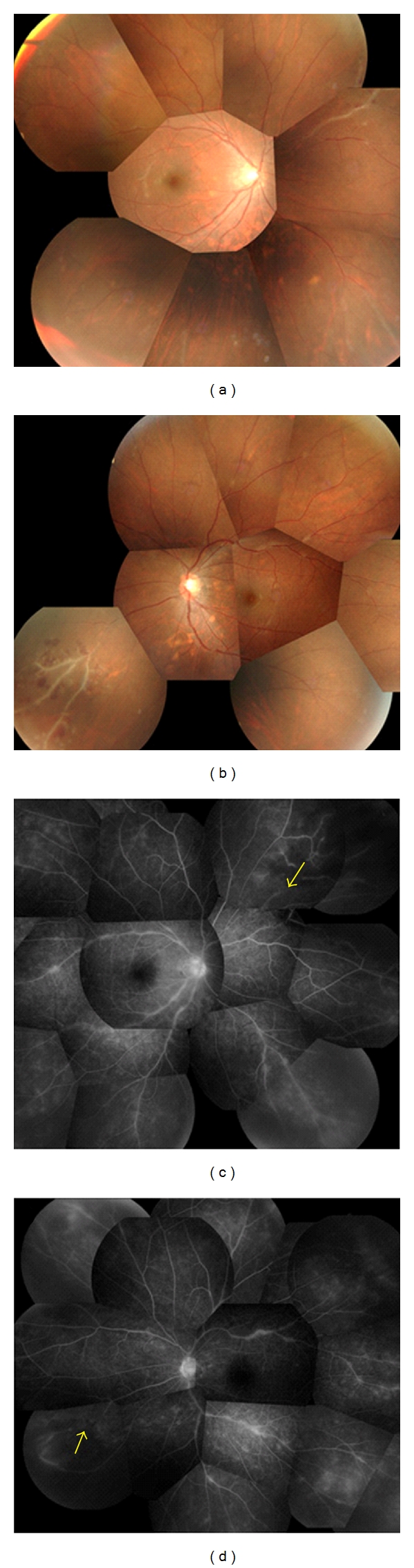
Composite photographs of the fundus and fluorescein angiograms of a patient with ocular sarcoidosis with an infarct in the medulla oblongata. (a) and (b) Infiltrates and ischemic areas can be seen. (c) and (d) Fluorescein angiograms of right and left eyes showing areas of nonperfusion (arrow) in both eyes.

A 36-year-old man reported blurred vision and redness of the conjunctiva in both eyes. He was referred to our hospital in January 2009, and our examination showed that his best-corrected visual acuity (BCVA) was 1.2 (20/20) in both eyes. Slit-lamp examination showed ciliary injection and keratic precipitates. Fundus examination demonstrated vasculitis in both eyes (Figures 1(a) and 1(b)).

Physical examination showed exudative erythema nodosum on his trunk and both thighs. Chest X-rays and CT scans demonstrated bilateral hilar and mediastinal lymph node swelling. Laboratory tests, including antibodies for rheumatoid factor, Sjögren syndrome, double-stranded DNA, and antinuclear antibodies, were negative. In addition, the serum antiotensin-converting enzyme level was high at 27.0 U/L (normal <20).

**Figure 2:**
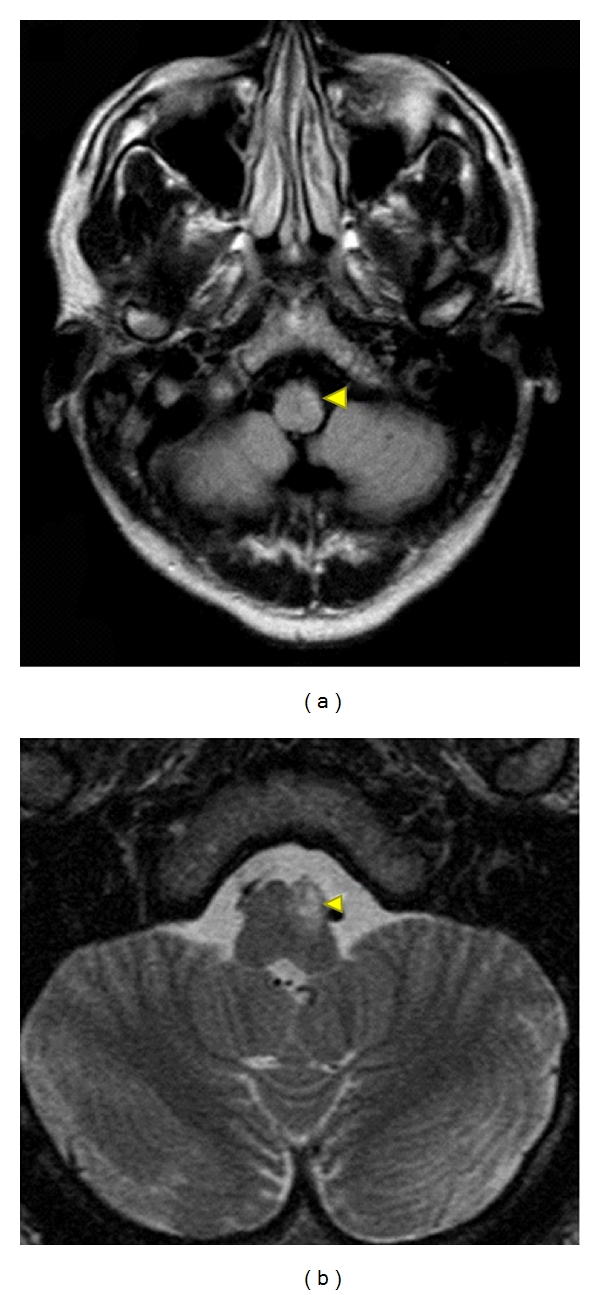
Images of the brain of a patient with an infarct in the medulla oblongata. (a) Left medulla oblongata encephalomalacia on axial T2-fluid-attenuated inversion image (arrow head). (b) Diffusion-weighted (TTt) image showing restricted diffusion in the left medulla oblongata (arrowhead).

**Figure 3:**
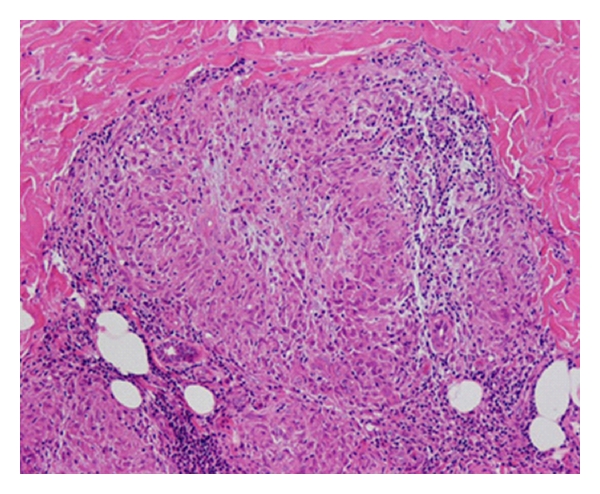
Histopathological section of skin biopsy. Nonnecrotizing granuloma with several multinucleated giant cells and epitheliod histiocytes can be seen. Hematoxylin-eosin ×250.

Two days after his first visit, he reported dizziness, speech disturbance, clumsiness of his right extremities, and gait disturbances. Neurological examinations showed left ptosis and miosis, hoarseness, dysphagia, right mild hemiparesis, left ataxia of his extremities, and truncal ataxia. He also had hypalgesia/hypotemperature on his right side. These findings suggested a left Wallenberg syndrome. Brain magnetic resonance imaging (MRI) showed an infarct on the left side of the medulla oblongata (Figure 2). Leptomeningeal enhancement was not observed on MRI. Studies of the cerebrospinal fluid showed normal glycorrhachia (74 mg/dL), mild lymphocyte elevation, and elevated protein at 69 mg/dL. These findings suggested inflammatory processes in the central nervous systems. Dermal examination/skin biopsy revealed granulomatous nodular dermatitis (Figure 3). 18F-FDG in the acute stage showed an upregulation of both hillar lymphadenitis in the mediastinum and ocular regions.

The patient was diagnosed with neurosarcoidosis with a brainstem infarct. Steroid pulse therapy was prescribed initially with 1000 mg infusion for three days and then slowly tapered. During the pulse therapy, the patient had laser photocoagulation of the nonperfused areas of the retina. The patient had an improvement of his symptoms after the steroid-pulse therapy.

At the last examination on February 2011, the patient was stable with no complications and no recurrence of the ocular and CNS sarcoidosis. And his BCVA was 20/20.

## 3. Discussion

Our findings showed that our patient had CNS sarcoidosis of the medulla oblongata, and steroid-pulse therapy was very effective in resolving the symptoms. The mechanism causing the brain infarction in neurosarcoidosis is not completely known but is thought to result from vasculitis of the small vessels, emboli, or inflammation of the large vessels [16, 17]. Granulomatous invasion into the blood vessel walls with disruption of the media and internal elastic laminae can cause infarctions of small vessels [18].

Although rare, neurosarcoidosis should be considered in cases of an acute stroke with restricted diffusion of unknown etiology in a young person, even if other stigmata of sarcoidosis are not present. Despite the common finding of vasculitis and microscopic infarction at autopsy, a stroke is usually due to vascular accidents in the cerebral hemisphere and is rarely the presenting sign of neurosarcoidosis (Table 1) [10, 11, 15]. The rarity of brainstem stroke is surprising given the propensity of basilar inflammation [10, 19]. To the best of our knowledge, our report is the first case of neurosarcoidosis with a brainstem infarction in the medulla oblongata.

Ocular sarcoidosis is not rare in Japan, and it has been found that sarcoidosis responds well to steroid therapy. But 10% of ocular sarcoidosis required periocular corticosteroids to treat the uveitis, and 13% of ocular sarcoidosis required systemic treatment for both the ocular and systemic disease [19]. In addition, 8% of patients required immunosuppressive therapy because steroids alone did not control the inflammation or the dose required to prevent a relapse of uveitis was too high [19]. Poor visual outcome ranged from 9% to 66% [19, 20].

The management of resistant uveitis associated with sarcoidosis is a serious therapeutic problem. The toxicity from steroids such as osteoporosis, femur head necrosis, infection, steroidgenic diabetes, corticosteroid-induced glaucoma, and steroid-induced psychosis is difficult to control. This is why clinicians resist the use of high-dose steroids for long periods. Thus, it is important to know that steroid-pulse therapy is effective in treating the ocular inflammation due to sarcoidosis. Because steroid-pulse therapy has fewer side-effect, we recommend steroid pulse therapy for ocular and CNS sarcoidosis.

## Figures and Tables

**Table 1:** Past reports which reported CNS sarcoidosis as brain ischemic stroke.

		Location	Eye symptom
Navi, 2009 [10]	35 M	Pons	None
	46 F	Pons	None
Hodge, 2007 [11]	36 f	Left frontal subcortical white matter	None
Brisman, 2006 [12]	41 m	Frontal and lobe	Left eye blindness
Nakagaki, 2004 [13]	75 m	Right parieto occipital lobes	Ocular sarcoidosis
Das, 1998 [14]	27 f	Infarction of left middle cerebral artery territory	None
Michotte, 1991 [15]	29 m	Multiple bilateral subcortical lesion	None
